# 2-[3,5-Dioxo-4-(pyridin-3-yl)piperazin-1-yl]acetic acid

**DOI:** 10.1107/S1600536812003455

**Published:** 2012-02-04

**Authors:** Mohammad Hossein Mosslemin

**Affiliations:** a Department of Chemistry, Yazd Branch, Islamic Azad University, PO Box 89195-155, Yazd, Iran

## Abstract

In the title compound, C_11_H_11_N_3_O_4_, the 3,5-dioxopiperazine ring adopts an envelope conformation, with the N atom connecting to the –CH_2_COOH group on the flap. In the crystal, mol­ecules are linked by O—H⋯N hydrogen bonds to produce a linear chain running along the *c* axis. π–π stacking is observed between parallel pyridine rings of adjacent mol­ecules, the centroid–centroid distance being 3.834 (2) Å.

## Related literature

For the pharmaceutical activity, see: Parcel (1961 ▶); Creighton *et al.* (1969 ▶); Hasinoff *et al.* (1998 ▶). For related structures, see: Hasinoff *et al.* (2004 ▶); Mancilla *et al.* (2002 ▶); Skrzypczak-Jankun *et al.* (1999 ▶); Hempel *et al.* (1981 ▶); Jolley *et al.* (1999 ▶); Liu *et al.* (1998 ▶); Davies *et al.* (1998 ▶); Smith *et al.* (1992 ▶).
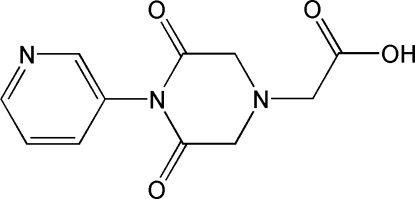


## Experimental

### 

#### Crystal data

C_11_H_11_N_3_O_4_
*M* *_r_* = 249.23
Orthorhombic, 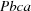

*a* = 12.762 (2) Å
*b* = 7.9495 (10) Å
*c* = 22.218 (3) Å
*V* = 2254.0 (6) Å^3^
*Z* = 8
Mo *K*α radiation
μ = 0.11 mm^−1^
*T* = 298 K
0.3 × 0.07 × 0.06 mm

#### Data collection

Bruker SMART 1000 CCD diffractometer
Absorption correction: multi-scan (*SADABS*; Bruker, 2001 ▶) *T*_min_ = 0.986, *T*_max_ = 0.996
5235 measured reflections
2181 independent reflections
1355 reflections with *I* > 2σ(*I*)
*R*_int_ = 0.082

#### Refinement

*R*[*F* ^2^ > 2σ(*F* ^2^)] = 0.083
*wR*(*F* ^2^) = 0.139
*S* = 1.13
2181 reflections
167 parameters
1 restraint
H atoms treated by a mixture of independent and constrained refinement
Δρ_max_ = 0.19 e Å^−3^
Δρ_min_ = −0.18 e Å^−3^

### 

Data collection: *SMART* (Bruker, 2007 ▶); cell refinement: *SAINT* (Bruker, 2007 ▶); data reduction: *SAINT*; program(s) used to solve structure: *SHELXTL* (Sheldrick, 2008 ▶); program(s) used to refine structure: *SHELXTL*; molecular graphics: *ORTEP-3 for Windows* (Farrugia, 1997 ▶); software used to prepare material for publication: *WinGX* (Farrugia, 1999 ▶).

## Supplementary Material

Crystal structure: contains datablock(s) global, I. DOI: 10.1107/S1600536812003455/xu5454sup1.cif

Structure factors: contains datablock(s) I. DOI: 10.1107/S1600536812003455/xu5454Isup2.hkl

Supplementary material file. DOI: 10.1107/S1600536812003455/xu5454Isup3.cml

Additional supplementary materials:  crystallographic information; 3D view; checkCIF report

## Figures and Tables

**Table 1:** Hydrogen-bond geometry (Å, °)

*D*—H⋯*A*	*D*—H	H⋯*A*	*D*⋯*A*	*D*—H⋯*A*
O4—H4⋯N1^i^	0.83 (3)	1.83 (3)	2.655 (4)	173 (5)

Symmetry code: (i) 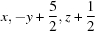.

**Fig. 1.**
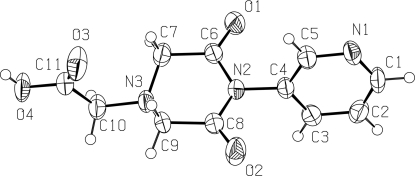
The molecular structure of the title molecule, with the atom-numbering scheme. Displacement ellipsoids are drawn at the 50% probability level.

**Fig. 2.**
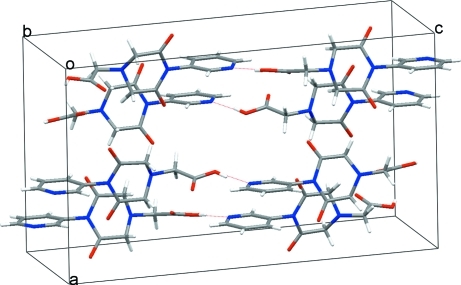
Unit-cell packing diagram for (I).

## supplementary crystallographic information

### Comment

The interest in derivatives of piperazine-2,6-dione has significantly increased because of their practical usage. Some piperazine-2,6-diones were found to have pharmaceutical activity (Parcel, 1961). In this regard, piperazine-2,6-diones have been studied for the anticancerous activity, an interaction with other anticancerous drugs and sensitivity to radiation (Creighton *et al.*, 1969; Hasinoff, *et al.*, 1998). In spite of the importance of these compounds, at present, only 12 crystal structures of piperazines are available in the literature, (Hasinoff *et al.*, 2004; Mancilla *et al.*, 2002; Skrzypczak-Jankun *et al.*, 1999; Hempel *et al.*, 1981; Jolley *et al.*, 1999; Liu *et al.*, 1998; Davies *et al.*, 1998 and Smith *et al.*, 1992). Here, we report the synthesis and characterization of the title compound, I.

In this molecule, the six membered piperazine-2,6-dionering has an envelope conformation with the N3 atom out of plane. The dihedral angle between the pyridine ring and the five-atom planar portion of the piperazine-2,6-dione ring is 77.9 (9)°. Also, the angle between mean plane containing acetic acid moiety and pyridine ring is 15.7 (8)°. As it is clear from figure 2, in the crystal packing of title molecule, the intermolecular O—H···N hydrogen bonds (Table 1) seem to be effective in the stabilization of the crystal structure.

### Experimental

A solution of 3-aminopyridine (0.94 gr, 0.01 mole) in 20 ml pyridine was added to a solution of 1.91 gr (0.01 mole) nitrilotriacetic acid in 20 ml pyridine. The resulting solution was stirred at 313 K for 1 h, then 2.6 ml triphenyl phosphite was added dropwise, and the reaction mixture was stirred at 373 K for 10 h and at ambient temperature for 48 h. the product was obtained by addition of cold water to oyridine solution. X-ray quality crystals were obtained by slow diffusion of diethyl ether into a CH_2_Cl_2_ solution at room temperature.

### Refinement

The carboxyl H atom was located in a difference Fourier map and refined isotropically. Other H atoms were positioned geometrically with C—H = 0.93 and 0.97 Å for aromatic H and methylene H atoms, respectively, and constrained to ride on their parent atoms with *U*_iso_(H) = 1.2*U*_eq_(C).

### Crystal data

**Table:**

C_11_H_11_N_3_O_4_	*F*(000) = 1040
*M**_r_* = 249.23	*D*_x_ = 1.469 Mg m^−^^3^
Orthorhombic, *P**b**c**a*	Mo *K*α radiation, λ = 0.71073 Å
Hall symbol: -P 2ac 2ab	Cell parameters from 5235 reflections
*a* = 12.762 (2) Å	θ = 2.4–26°
*b* = 7.9495 (10) Å	µ = 0.11 mm^−^^1^
*c* = 22.218 (3) Å	*T* = 298 K
*V* = 2254.0 (6) Å^3^	Block, colorless
*Z* = 8	0.3 × 0.07 × 0.06 mm

### Data collection

**Table:**

Bruker SMART 1000 CCD diffractometer	1355 reflections with *I* > 2σ(*I*)
Graphite monochromator	*R*_int_ = 0.082
φ and ω scans	θ_max_ = 26.0°, θ_min_ = 2.4°
Absorption correction: multi-scan (*SADABS*; Bruker, 2001)	*h* = −15→13
*T*_min_ = 0.986, *T*_max_ = 0.996	*k* = −9→8
5235 measured reflections	*l* = −27→18
2181 independent reflections	

### Refinement

**Table:**

Refinement on *F*^2^	Primary atom site location: structure-invariant direct methods
Least-squares matrix: full	Secondary atom site location: difference Fourier map
*R*[*F*^2^ > 2σ(*F*^2^)] = 0.083	Hydrogen site location: inferred from neighbouring sites
*wR*(*F*^2^) = 0.139	H atoms treated by a mixture of independent and constrained refinement
*S* = 1.13	*w* = 1/[σ^2^(*F*_o_^2^) + (0.0234*P*)^2^ + 2.3505*P*] where *P* = (*F*_o_^2^ + 2*F*_c_^2^)/3
2181 reflections	(Δ/σ)_max_ = 0.003
167 parameters	Δρ_max_ = 0.19 e Å^−^^3^
1 restraint	Δρ_min_ = −0.18 e Å^−^^3^

### Special details

**Table:**

Geometry. All e.s.d.'s (except the e.s.d. in the dihedral angle between two l.s. planes) are estimated using the full covariance matrix. The cell e.s.d.'s are taken into account individually in the estimation of e.s.d.'s in distances, angles and torsion angles; correlations between e.s.d.'s in cell parameters are only used when they are defined by crystal symmetry. An approximate (isotropic) treatment of cell e.s.d.'s is used for estimating e.s.d.'s involving l.s. planes.
Refinement. Refinement of *F*^2^ against ALL reflections. The weighted *R*-factor *wR* and goodness of fit *S* are based on *F*^2^, conventional *R*-factors *R* are based on *F*, with *F* set to zero for negative *F*^2^. The threshold expression of *F*^2^ > σ(*F*^2^) is used only for calculating *R*-factors(gt) *etc*. and is not relevant to the choice of reflections for refinement. *R*-factors based on *F*^2^ are statistically about twice as large as those based on *F*, and *R*- factors based on ALL data will be even larger.

### Fractional atomic coordinates and isotropic or equivalent isotropic displacement parameters (Å^2^)

**Table:**

	*x*	*y*	*z*	*U*_iso_*/*U*_eq_	
C1	0.6306 (3)	0.9688 (5)	0.48064 (15)	0.0465 (10)	
H1	0.6305	0.9635	0.4388	0.056*	
C2	0.6268 (3)	0.8204 (5)	0.51293 (17)	0.0468 (10)	
H2	0.624	0.7174	0.4932	0.056*	
C3	0.6274 (3)	0.8274 (5)	0.57515 (16)	0.0409 (9)	
H3	0.6243	0.7296	0.598	0.049*	
C4	0.6326 (3)	0.9824 (4)	0.60224 (14)	0.0352 (8)	
C5	0.6357 (3)	1.1239 (5)	0.56714 (15)	0.0420 (9)	
H5	0.6388	1.2282	0.5861	0.05*	
C6	0.5383 (3)	1.0558 (4)	0.69368 (15)	0.0353 (8)	
C7	0.5406 (3)	1.0795 (5)	0.76080 (15)	0.0429 (9)	
H7A	0.5573	1.1957	0.7701	0.051*	
H7B	0.4721	1.0547	0.7774	0.051*	
C8	0.7256 (3)	0.9787 (5)	0.69787 (16)	0.0396 (9)	
C9	0.7218 (3)	1.0056 (5)	0.76503 (15)	0.0422 (9)	
H9A	0.7722	0.9324	0.7845	0.051*	
H9B	0.7404	1.1211	0.7742	0.051*	
C10	0.6095 (4)	0.9576 (4)	0.85320 (15)	0.0459 (10)	
H10A	0.6585	0.8723	0.8668	0.055*	
H10B	0.5396	0.9184	0.8629	0.055*	
C11	0.6299 (3)	1.1167 (5)	0.88859 (15)	0.0403 (9)	
N1	0.6345 (3)	1.1197 (4)	0.50719 (13)	0.0436 (8)	
N2	0.6320 (2)	1.0005 (4)	0.66694 (11)	0.0347 (7)	
N3	0.6179 (2)	0.9703 (3)	0.78789 (11)	0.0368 (7)	
O1	0.4629 (2)	1.0852 (4)	0.66345 (12)	0.0542 (8)	
O2	0.8058 (2)	0.9448 (4)	0.67151 (13)	0.0665 (9)	
O3	0.6582 (3)	1.2471 (3)	0.86656 (12)	0.0666 (9)	
O4	0.6119 (3)	1.0957 (4)	0.94599 (11)	0.0555 (8)	
H4	0.624 (4)	1.184 (3)	0.9643 (18)	0.075 (16)*	

### Atomic displacement parameters (Å^2^)

**Table:**

	*U*^11^	*U*^22^	*U*^33^	*U*^12^	*U*^13^	*U*^23^
C1	0.046 (2)	0.073 (3)	0.0202 (16)	0.001 (2)	−0.0019 (16)	−0.0060 (17)
C2	0.047 (2)	0.054 (2)	0.039 (2)	0.005 (2)	−0.0012 (18)	−0.0145 (18)
C3	0.048 (2)	0.040 (2)	0.0349 (19)	0.003 (2)	−0.0048 (18)	0.0003 (15)
C4	0.0376 (19)	0.042 (2)	0.0254 (17)	−0.0002 (17)	−0.0022 (15)	−0.0012 (15)
C5	0.050 (2)	0.045 (2)	0.032 (2)	−0.0020 (19)	−0.0027 (17)	−0.0003 (16)
C6	0.043 (2)	0.0343 (18)	0.0291 (17)	0.0007 (17)	−0.0040 (15)	0.0005 (15)
C7	0.042 (2)	0.057 (2)	0.0298 (19)	0.002 (2)	0.0034 (16)	−0.0072 (17)
C8	0.039 (2)	0.049 (2)	0.0312 (19)	0.0054 (18)	−0.0010 (16)	0.0000 (16)
C9	0.048 (2)	0.055 (2)	0.0230 (17)	0.011 (2)	−0.0047 (15)	0.0000 (16)
C10	0.069 (3)	0.041 (2)	0.0267 (19)	−0.002 (2)	0.0033 (18)	−0.0004 (15)
C11	0.047 (2)	0.047 (2)	0.0272 (18)	0.0006 (19)	0.0003 (16)	−0.0007 (16)
N1	0.0453 (19)	0.059 (2)	0.0262 (16)	−0.0008 (17)	−0.0003 (14)	0.0072 (14)
N2	0.0394 (16)	0.0410 (17)	0.0237 (14)	0.0048 (14)	0.0025 (12)	0.0010 (12)
N3	0.053 (2)	0.0360 (15)	0.0218 (14)	−0.0003 (15)	0.0027 (13)	0.0013 (11)
O1	0.0466 (16)	0.077 (2)	0.0385 (15)	0.0163 (16)	−0.0113 (12)	−0.0093 (14)
O2	0.0398 (16)	0.121 (3)	0.0391 (17)	0.0152 (18)	−0.0009 (13)	−0.0150 (17)
O3	0.112 (3)	0.0494 (16)	0.0379 (15)	−0.0274 (19)	0.0120 (17)	−0.0063 (14)
O4	0.091 (2)	0.0530 (18)	0.0222 (13)	−0.0052 (18)	0.0078 (14)	−0.0028 (12)

### Geometric parameters (Å, º)

**Table:**

C1—N1	1.338 (5)	C7—H7A	0.97
C1—C2	1.381 (5)	C7—H7B	0.97
C1—H1	0.93	C8—O2	1.210 (4)
C2—C3	1.383 (5)	C8—N2	1.389 (4)
C2—H2	0.93	C8—C9	1.508 (5)
C3—C4	1.373 (5)	C9—N3	1.446 (4)
C3—H3	0.93	C9—H9A	0.97
C4—C5	1.369 (5)	C9—H9B	0.97
C4—N2	1.445 (4)	C10—N3	1.459 (4)
C5—N1	1.332 (4)	C10—C11	1.511 (5)
C5—H5	0.93	C10—H10A	0.97
C6—O1	1.197 (4)	C10—H10B	0.97
C6—N2	1.406 (4)	C11—O3	1.202 (4)
C6—C7	1.504 (4)	C11—O4	1.307 (4)
C7—N3	1.446 (4)	O4—H4	0.829 (10)
			
N1—C1—C2	122.5 (3)	O2—C8—C9	122.5 (3)
N1—C1—H1	118.7	N2—C8—C9	116.4 (3)
C2—C1—H1	118.7	N3—C9—C8	110.4 (3)
C1—C2—C3	119.0 (4)	N3—C9—H9A	109.6
C1—C2—H2	120.5	C8—C9—H9A	109.6
C3—C2—H2	120.5	N3—C9—H9B	109.6
C4—C3—C2	118.3 (4)	C8—C9—H9B	109.6
C4—C3—H3	120.8	H9A—C9—H9B	108.1
C2—C3—H3	120.8	N3—C10—C11	116.6 (3)
C5—C4—C3	119.3 (3)	N3—C10—H10A	108.1
C5—C4—N2	119.0 (3)	C11—C10—H10A	108.1
C3—C4—N2	121.7 (3)	N3—C10—H10B	108.1
N1—C5—C4	123.2 (4)	C11—C10—H10B	108.1
N1—C5—H5	118.4	H10A—C10—H10B	107.3
C4—C5—H5	118.4	O3—C11—O4	124.1 (4)
O1—C6—N2	120.5 (3)	O3—C11—C10	124.2 (3)
O1—C6—C7	123.3 (3)	O4—C11—C10	111.8 (3)
N2—C6—C7	116.2 (3)	C5—N1—C1	117.7 (3)
N3—C7—C6	110.6 (3)	C8—N2—C6	124.2 (3)
N3—C7—H7A	109.5	C8—N2—C4	118.4 (3)
C6—C7—H7A	109.5	C6—N2—C4	117.1 (3)
N3—C7—H7B	109.5	C7—N3—C9	111.2 (3)
C6—C7—H7B	109.5	C7—N3—C10	113.9 (3)
H7A—C7—H7B	108.1	C9—N3—C10	115.5 (3)
O2—C8—N2	121.1 (3)	C11—O4—H4	110 (3)
			
N1—C1—C2—C3	−0.2 (6)	O2—C8—N2—C4	−0.3 (6)
C1—C2—C3—C4	−0.5 (6)	C9—C8—N2—C4	177.6 (3)
C2—C3—C4—C5	0.8 (6)	O1—C6—N2—C8	173.7 (3)
C2—C3—C4—N2	179.1 (3)	C7—C6—N2—C8	−4.6 (5)
C3—C4—C5—N1	−0.4 (6)	O1—C6—N2—C4	0.4 (5)
N2—C4—C5—N1	−178.7 (3)	C7—C6—N2—C4	−177.9 (3)
O1—C6—C7—N3	154.6 (3)	C5—C4—N2—C8	−99.6 (4)
N2—C6—C7—N3	−27.2 (5)	C3—C4—N2—C8	82.1 (5)
O2—C8—C9—N3	−154.6 (4)	C5—C4—N2—C6	74.1 (4)
N2—C8—C9—N3	27.5 (5)	C3—C4—N2—C6	−104.2 (4)
N3—C10—C11—O3	−4.7 (6)	C6—C7—N3—C9	60.3 (4)
N3—C10—C11—O4	174.7 (4)	C6—C7—N3—C10	−167.1 (3)
C4—C5—N1—C1	−0.4 (6)	C8—C9—N3—C7	−60.4 (4)
C2—C1—N1—C5	0.7 (6)	C8—C9—N3—C10	167.8 (3)
O2—C8—N2—C6	−173.6 (4)	C11—C10—N3—C7	−64.7 (5)
C9—C8—N2—C6	4.4 (5)	C11—C10—N3—C9	65.9 (5)

### Hydrogen-bond geometry (Å, º)

**Table:**

*D*—H···*A*	*D*—H	H···*A*	*D*···*A*	*D*—H···*A*
O4—H4···N1^i^	0.83 (3)	1.83 (3)	2.655 (4)	173 (5)

Symmetry code: (i) *x*, −*y*+5/2, *z*+1/2.
